# Mr. Kynion, on Swelled Legs

**Published:** 1801-03

**Authors:** F. Kynion

**Affiliations:** London


### *To the Editors of the Medical and Phyſical Journal.*

Gentlemen,

Among the various diſeaſes which afflict our ſpecies, I think there is one which has not ſufficiently occupied the attention of medical practitioners; I mean that of ſwelled legs. It is preſumed that this diſeaſe may ſubſiſt where the viſcera are ſound. It is not an unfrequent complaint with men advancing in years, and it is alſo a diſorder which ſometimes affects young men of libidinous habits, evidently proceeding from mere relaxation. It is therefore ſubmitted for the conſideration of our ſurgeons, if it might not be uſeful in obſtinate caſes, to render more general the practice of bandages, or of iſſues, or ſome other more effectual kind of drain, for the diſcharge of the water, and for the prevention of a future accumulation. The writer, although not a practitioner, yet having been deſigned and educated for the profeſſion, he is fully aware of the uſual objections in ſuch caſes. He does not think that iſſues, or any ſimilar plan for diſcharging the water, would produce mortification. In an incipient ſtate of the diſeaſe, there is commonly ſufficient vigour remaining in the conſtitution to reſiſt that tendency; and that vigour would be in a great degree preſerved, by preventing the tone of the part from being injured by diſtention. It ſhould alſo be conſidered, that where the viſcera are ſound, the water is not always in a very abundant quantity; but in a quantity ſufficient to ſhorten the lives of many; and not only to ſhorten, but by producing an oppreſſion upon the ſtomach, and other unpleaſant feelings, to render their lives very uncomfortable: Whereas, could this accumulation of fluid by any means be obviated, ſuch perſons might enjoy many years of happineſs and eaſe.

